# Congenital glioblastoma – prenatal diagnosis becoming a diagnostic challenge after birth: a case report

**DOI:** 10.1515/crpm-2023-0008

**Published:** 2023-09-04

**Authors:** Orzeł M. Maria, Pruszek K. Weronika, Borek-Dzięcioł Beata, Głuszczak-Idziakowska Ewa, Kociszewska-Najman Bożena

**Affiliations:** Medical University of Warsaw, Warsaw, Masovian Voivodeship, Poland; Department of Neonatology and Rare Diseases, Children's Clinical Hospital, University Clinical Centre of the Medical University of Warsaw, Warsaw, Poland

**Keywords:** newborn, intraventricular hemorrhage, central nervous system, tumor, congenital, glioblastoma

## Abstract

**Objectives:**

The incidence of congenital brain tumors is estimated at 1.1–3.6 per 100.000 live births, accounting for 0.5–2 % of all cancers in the pediatric population. Congenital gliomas account for 3.1–8.9 % of all congenital brain tumors and are cancers with a poor prognosis. The rate of stillbirth and death on the first day of life reaches 29 %; 38 % die within the first week, and 56 % die within the first two months. The average length of survival is two years.

**Case presentation:**

In the 29th week of pregnancy, a female fetus was diagnosed with intracranial hemorrhage complicated by hydrocephalus. Postnatal brain MRI imaging showed a solid proliferative lesion of the left hemisphere with dilatation of the ventricular system. Brown cerebrospinal fluid was collected during the puncture of the left lateral ventricle to reduce hydrocephalus. No tumor cells were detected by cytology. Due to increasing hydrocephalus, the patient was qualified for Rickham reservoir implantation. On day 27th, a craniotomy was performed to determine the etiology of recurrent prenatal intraventricular bleeding. During surgery, the bleeding mass raised the suspicion of neoplasm—histopathological examination of the retrieved tissue diagnosed WHO stage IV malignant glioma. The patient died at 8 months of age.

**Conclusions:**

Prenatal diagnosis of an abnormal structure in the fetal brain remains a diagnostic challenge in neonates. Glioblastoma is a rare neoplasm with a poor prognosis.

## Introduction

Although congenital brain tumors rarely occur in newborns as an isolated finding, they can be overlooked by ultrasound and diagnosed as intraventricular hemorrhage, hypertensive hydrocephalus, and macrocephaly as secondary findings [1]. Therefore, more sensitive neuroimaging studies are helpful in cases of suspected CNS proliferative processes.

The onset of congenital brain tumors is usually in the third trimester, and very rarely can be found in early pregnancy [2]. Among congenital brain tumors diagnosed in the perinatal period, glioblastoma is among the most often diagnosed tumors with a poor prognosis [3]. If prenatally diagnosed, glioblastoma may cause intrauterine death in 29 % of fetuses, while postnatally the prognosis is even worse with 38 % of infants dying in the early neonatal period, and 56 % dying in the first two months of life [3]. The average survival time after the diagnosis of glioblastoma in the perinatal period is about two years [3].

We are presenting a neonate who has been prenatally diagnosed by ultrasound and magnetic resonance imaging (MRI) with intracranial hemorrhage and posthemorrhagic hypertensive hydrocephalus which occurred to be secondary to glioblastoma diagnosed by MRI and histopathology postnatally.

## Case presentation

A fetal ultrasound followed by prenatal MRI at the 29th week of pregnancy in a 38-year women gravida 5 para 3, showed an intracerebral hemorrhage progressing to posthemorrhagic hypertensive hydrocephalus with cystic and solid structures in the cerebral cortex considered to be extensive leukomalacia.

The course of the pregnancy, except for abnormalities on prenatal examination, was uneventful. On the 29th week of gestation, the mother received a course of corticosteroids in case of indication for emergency premature birth. Due to intracranial hemorrhage and hypertensive hydrocephalus with unclear intracerebral lesions emergency cesarean section was performed at 36+0 weeks of gestation.

The cardiorespiratory stable baby girl was born with a birth weight of 3240 g (on Fenton growth calculator available at https://peditools.org/fenton2013/index.php Z-score 1.36 – which is large for 36+0 GA), head circumference 38 cm (Fenton growth calculator Z-score 3.92), body length 53 cm (Fenton growth chart growth calculator Z-score 2.55), and Apgar score was 10/7/10 at 1, 5, and 10 min of life.

The baby-girl was transferred to the Neonatal Intensive Care Unit (NICU) for follow-up. On physical examination after birth, only a large head with irritability and variable muscle tone has been found.

During the observation baby-girl developed at the age of several hours sudden automatic movements of the limbs which ceased after the introduction of phenobarbital followed by phenytoin. From the 2nd day of life due to inadequate respiratory effort and decreased oxygen saturation (SpO2), nasal continuous airway pressure(N-CPAP) with air was used for five days followed by the occasionally increased fraction of inspired oxygen (FiO2) up to 25 %. The lower oxygen saturation was caused by anemia secondary to intracranial hemorrhage needing two transfusions of red blood cell concentrate.

Besides anemia, all other laboratory findings have been uneventful as were the ocular fundus and cardiac ultrasound. By usual neonatal screening tests, some rare metabolic and genetic conditions have been excluded. Bacterial and TORCH infections have been excluded by appropriate blood tests.

**Figure 1:**
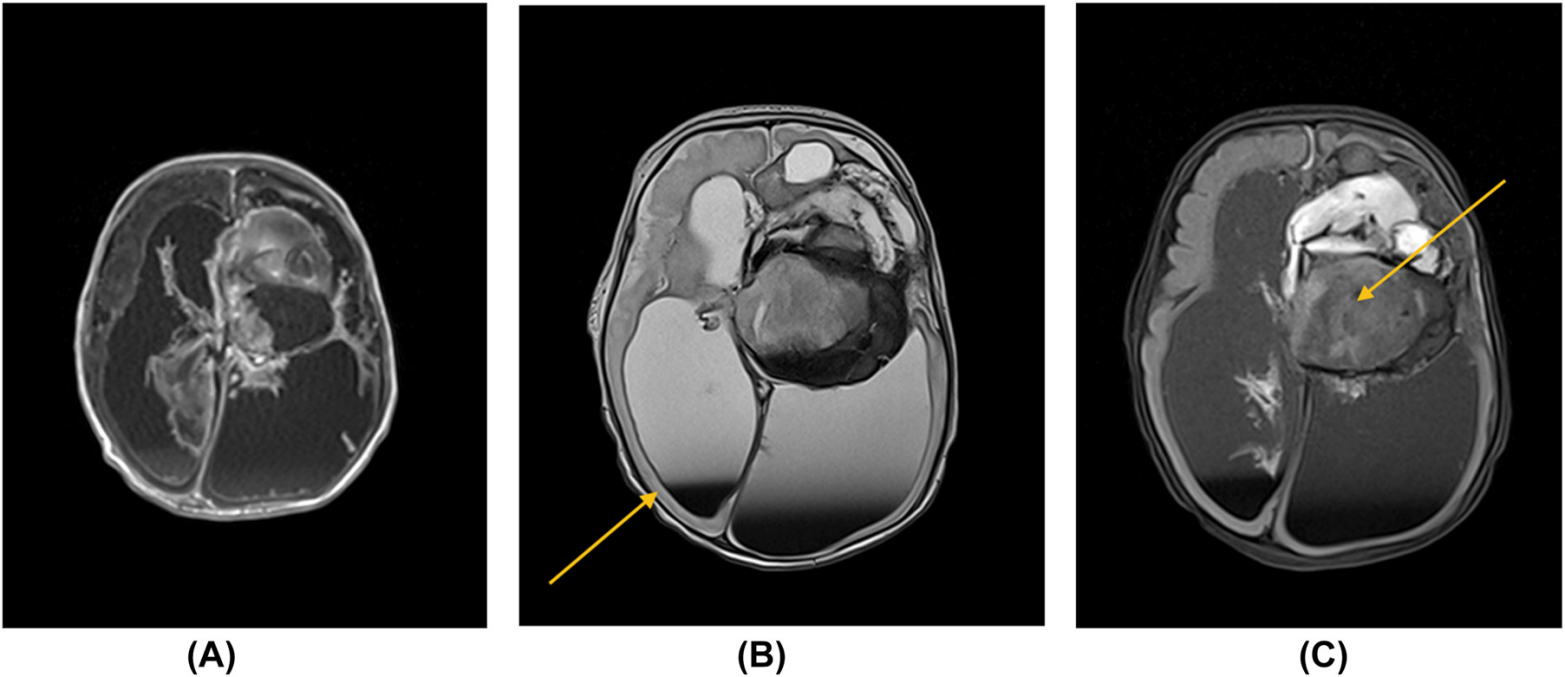
Newborn head MRI at 2nd day of life (A–C). In the image B an arrow marks the brain tissue. The tumor mass is marked with an arrow in image C.

Postnatal brain ultrasound revealed the same results as found on prenatal ultrasound and MRI. Postnatal MRI on the 2nd day of life revealed a focal subcortical lesion of the left lateral frontal horn measuring 56 × 49 × 57 mm with heterogeneous signal intensity (Figure 1). Contrast enhancement of the mass was visualized with the displacement of medial structures and third ventricle to the right by 11 mm. The width of the third ventricle was decreased. The thickness of the cortical brain layer in both frontal regions was 11 mm, in the parietal region it measured up to 6 mm, and in the right occipital region, it was only 4 mm, while in the left occipital region cortical layer was mostly absent.

Due to seizure-like episodes, the amplitude-integrated EEG continuous monitoring was initiated on the 2nd day of life which revealed frequent seizure patterns confirmed by conventional EEG recording with bilateral complexes of sharp and slow wave brain activity mostly lasting from several seconds to one minute.

The condition of the baby has been deteriorating in terms of increasing intraventricular pressure, which was the indication for the transfontanellar ventricular puncture of the lateral ventricles on the 14th and 21st day of life when 25 and 40 mL of the brownish cerebrospinal fluid (CSF) with high protein and the low number of cells was obtained. As the CSF cultures remained negative, due to increasing hydrocephalus shunt procedure with the insertion of the Rickham reservoir was performed on the 21st day of life.

**Figure 2:**
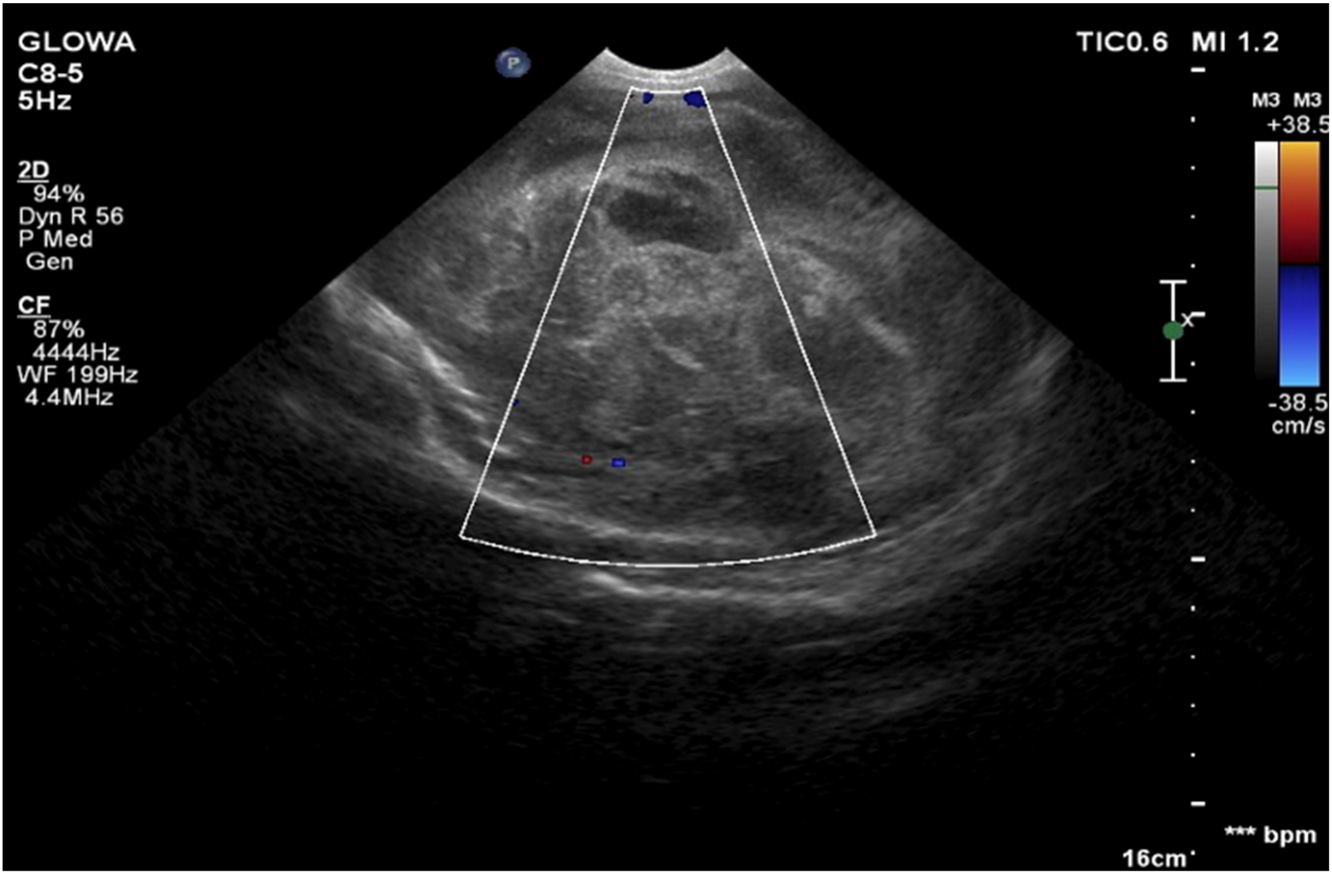
Trans-temporal ultrasound of a newborn with glioblastoma on the lateral plane. Visible lack of flow within the tumor on Doppler examination.

After the puncture, a transcranial ultrasound visualized an extensive mass 6 cm in diameter in the left thalamus (Figure 2). The lesion progressed to the right thalamus, closing the Monro orifices, and pressing the brainstem and other adjacent structures.

On the 27th day of life an open biopsy of the structure belonging to the left thalamic region was performed, and clots were removed from the left lateral ventricle.

Results of histopathological examination revealed malignant glioblastoma WHO grade IV with micrometastases, hemorrhages, and numerous mitoses. Immunohistochemistry (IHC) results: GFAP+, S100+, CD56+, Ki67 high, based on which diagnosis for *H3K27M* mutation was recommended.

Based on the overall clinical picture, imaging findings (two MRIs of the head), and mainly on histopathological findings, chemotherapy was initially considered. Ultimately, the neonate was not qualified for chemotherapy, which could have caused severe complications without benefit. However, it was possible to readmit the patient in case of favorable evolution of CNS bleeding. Palliative care has been considered with the continuation of phenobarbital, analgesics (methadone, paracetamol), and vitamins.

After consulting with oncologists, neurosurgeons, and the parents, the child was transferred to a home hospice for palliative care. At discharge on the 57th day of life, the girl’s condition was stable without features of increased intracranial pressure, bottle-fed, with appropriate weight gain. At the discharge, her weight was 5280 g (96th percentile), length 58 cm (98th percentile), and head circumference 41 cm (100th percentile). The same palliative treatment continued till the end of her life at eight months.

## Discussion

Brain tumors are sporadic under the age of 6 months [2]. According to the literature, congenital brain tumors are diagnosed prenatally or by two months of age. Seldomly glioblastoma can be diagnosed in the third trimester of pregnancy, while the diagnosis is very rare in early pregnancy. In our case, the tumor was presented as an intracerebral hemorrhage on prenatal examination at the 29th week of gestation. According to the literature, only 7 % of congenital brain gliomas are diagnosed prenatally [4]. However, due to the high mortality rate, the diagnosis is occasionally made at autopsy [3].

The most common mutations of high-grade tumors (WHO grades III and IV) in the pediatric population – are histone mutations (*H3F3A – H3K27 M or H3.3G34R/V*) and amplifications (*PDGFRA in EGFR, MYC, MYCN, and MDM4.27*). However, the *H3K27 M* mutation found in our case has appeared in only 6 % of brain tumors in the literature [5]. In addition, there are often foci of abundant vascular proliferation in malignant glioblastoma.

The distorted vessels of a rapidly growing tumor are prone to damage. Consequently, they often bleed at different times [6]. Brain tumors can therefore be complicated by bleeding diagnosed on prenatal or postnatal ultrasound. Such ultrasound findings on the fetal brain may raise suspicion of congenital vascular malformation. Symptomatic intracranial hemorrhage in neonates born at term is rare and usually may be caused by perinatal trauma, coagulation disorders, or hypoxia. In fetuses with intracranial bleeding, brain tumor is a rare diagnosis, usually overlooked and mostly found at autopsy [7]. The suspicion of brain neoplasm in our patient was prenatally overlooked on ultrasound and MRI images which showed only intracranial hemorrhage and suspected hypoxic-ischemic encephalopathy.

MRI was the most important postnatal diagnostic imagining together with the transfontanellar lateral ventricle puncture and cytology on the 37th postnatal day, which enabled proper diagnosis of fetal glioblastoma confirmed by open surgery biopsy of the mass.

Presence of tumor cells of glioblastoma in CSF not found in our patient, can be found in only 3 % of symptomatic patients with the condition [8].

The symptoms of glioblastoma described in the literature and present in our patient were: macrocephaly, hydrocephaly, abnormal muscle tone, and seizures. Other symptoms that may occur in congenital glioblastoma which were not observed in our patient, include vomiting, bulging fontanel, and some focal neurological signs [9].

Treatment options for congenital glioblastoma are limited. Due to the small number of reported cases and many patients dying early, there are no guidelines for chemotherapy or radiation in patients with congenital glioblastoma, while palliative and symptomatic treatment is available [2, 3]. Surgery remains the primary treatment option for brain tumors in newborns, regardless of the stage of the tumor [10]. High malignancy glioblastoma (WHO grades III and IV), like the one in our patient, is treated with surgery and radiation therapy. Possible treatment options after resection include chemotherapy and/or adjuvant subsequent radiotherapy [2, 3]. Unfortunately, only 40 % of children are qualified for chemotherapy after tumor resection [2, 3]. Our patient was not qualified for chemotherapy due to recurrent intraventricular bleeding. Excluding infants with glioblastoma who die in the first days of life, the average survival time of those infants is three years with treatment vs. five weeks without treatment [2, 3]. Our patient after surgery lived for eight months.

Despite the large tumor mass and significantly reduced brain volume, the girl had preserved visual fixation. In addition, the sucking and grasping reflexes were present with no limb paresis. During her stay in the hospital, she received treatment with analgesics, anticonvulsants, vitamins. Eventually, a ventriculoperitoneal valve was implanted to decompress the hydrocephalus. She also had neurosurgery and was transfused twice with red blood cell concentrate for anemia.

Ultimately, genetic testing on our patient was not performed, as she was not qualified for targeted chemotherapy treatment due to her unfavorable clinical condition, which would make the result of genetic testing only of scientific importance.

## Conclusions

Intracranial bleeding caused by glioblastoma detected on a prenatal ultrasound scan may be the first sign of the tumor which may be easily overlooked. Recognizing glioblastoma prenatally may be of high importance for fetal patients who should be referred to a properly equipped perinatal center where they can get proper medical care. Adequate prenatal neuroimaging options like MRI together with postnatal diagnostic procedures including tumor biopsy are crucial for the diagnosis of glioblastoma. Regardless of proper diagnosis and treatment, in most cases of congenital glioblastoma, the mortality is high and the prognosis is unfavorable. In many cases, like ours, the only option which could be offered is palliative care.
